# Supplementary material for: Antimicrobial Potential of Betulinic Acid and Investigation of the Mechanism of Action against Nuclear and Metabolic Enzymes with Molecular Modeling
Source: Pathogens. 2023 Mar 13;12(3):449. doi: 10.3390/pathogens12030449 (PMC10058483; doi:10.3390/pathogens12030449)
Supplement: Supplementary file 1 [file pathogens-12-00449-s001.zip › pathogens-2150293-supplementary.pdf]

Article type: Original article

IN VITRO EVALUATION OF THE ANTIMICROBIAL POTENTIAL OF BETULINIC ACID AND ANALYSIS OF MECHANISMS OF ACTION WITH MOLECULAR MODELING

Gabriela Cristina Soares Rodrigues[1], Mayara dos Santos Maia[1], Thalisson Amorim de Souza[2], Edeltrudes de Oliveira Lima[3], Luiz Eduardo C. G. dos Santos[3], Shellygton Lima Silva[3], Marcelo Sobral da Silva[2], José Maria Barbosa Filho[2]; Valnês da S. Rodrigues-Junior[4,5], Luciana Scotti[1], Marcus Tullius Scotti[1].

[1]Laboratory of Cheminformatics, Program of Natural and Synthetic Bioactive Products (PgPNSB), Health Sciences Center, Federal University of Paraíba, João Pessoa-PB, Brazil

[2] Multi-User Characterization and Analysis Laboratory, Program of Natural and Synthetic Bioactive Products (PgPNSB), Health Sciences Center, Federal University of Paraíba, João Pessoa-PB, Brazil.

[3]Program of Natural and Synthetic Bioactive Products (PgPNSB), Health Sciences Center, Federal University of Paraíba, 58051-900, João Pessoa-PB, Brazil

[4]National Institute of Science and Technology in Tuberculosis (INCT-TB), Brazil

[5]Program of Biotechnology, Center for Biotechnology, Federal University of Paraíba, Campus I, Cidade Universitária, João Pessoa 58051-900, PB, Brazil.

Correspondence: luciana.scotti@gmail.com; Tel.: +55 83 99624 5075

Table S1. Access codes of selected protein sequences obtained from the GenBank database.

	DNA gyrase	beta-lactamase	PBP
Staphylococcus aureus	BAR07518.1	KSA62127.1	BBA24197.1
S. epidermidis	KSZ68723.1	RUN10939.1	EJE17909.1
Pseudomonas aeruginosa	OHQ53593.1	KJJ11413.1	TEF07458.1
Escherichia coli	KHJ14714.1	KXG94406.1	ARB43848.1
Mycobacterium tuberculosis	AIH53390.1	KMY17933.1	QRX86232.1
	CYP51	SAP-2	DHFR
Candida albicans	AIQ80983.1	AAM21051.1	AAC05610.1
C. tropicalis	QXT50470.1	AAD33216.1	XP_002550914.1
C. glabrata	KTB23913.1	-	KTB19277.1

Aspergillus flavus	QOR32183.1	-	RMZ41744.1
Penicillium citrinum	-	-	-
Trichophyton rubrum	-	-	OAL62276.1
Microsporum canis	-	-	EEQ31600.1

Table S2. PDB codes of template proteins and degree of identity proteins of interest.

Species	Protein	ID PDB molde	Identity
	DNA gyrase	5NPP	93.83%
S. epidermidis	beta-lactamase	1XA1	94.90%
	PBP	3VSL	83.18%
	SAP-2	2H6S	51.20%
C. tropicalis	DHFR	1AOE	81.25%
A. flavus	CYP51	6CR2	64.03%
Trichophyton rubrum	DHFR	6DRS	48.97%

Table S3. Percentage of amino acids present in the allowed and favored regions of the Ramachandran plot for each model and percentage of the degree of compatibility between the 3D structure and the 1D amino acid sequence, based on the models generated by Verify 3D.

Species	Protein	Ramachandran	Verify 3D
	DNA gyrase	99.3%	89.63%
S. epidermidis	beta-lactamase	97.8%	100%
	PBP	98.5%	90.52%
	SAP-2	98.7%	93.15%
C. tropicalis	DHFR	100%	92.23%
A. flavus	CYP51	98.8%	93.02%
Trichophyton rubrum	DHFR	100%	97.51%

Table S4. RMSD values for the proteins selected in the study.

Species	Protein	PDB ligand	RMSD
	DNA gyrase	E32	-
S. aureus	beta-lactamase	benzylpenicillin	0.29
	PBP	cefotaxime	0.30
	DNA gyrase	EZ6	0.25
P. aeruginosa	beta-lactamase	imipenem	0.32
	PBP	imipenem	0.44
	DNA gyrase	6G9	0.25
E. coli	beta-lactamase	ácido borônico	0.23
	PBP	-	-
	DNA gyrase	MDP	0.29
M. tuberculosis	beta-lactamase	amoxicilin	0.42
	PBP	ampicilin	0.30
	CYP	posaconazole	0.83
C. albicans	SAP-2	benzamidine	0.19

	DHFR	N22	0.61
C. tropicalis	CYP	-	-
C. glabrata	CYP	-	-
A. flavus	DHFR	H8A	0.32

Figure S1. Alignment of DNA gyrase enzyme sequences from selected bacteria in the study. The gray regions correspond to non-similar and non-identical amino acids. The red regions correspond only to identical amino acids. The yellow regions are similar amino acids. The black boxes represent the active site amino acids.

```
M. tuberculosis  MTDTTLPPDDSLDRIEPVDIQQEMQRSYIDYAMSVIVGRALPEVRDGLKPVHRRVLYAMF  60
S. aureus        MKEELLMAELPQSRINERNITSEMRESFLDYAMSVIVARALPDVRDGLKPVHRRILYGLN  60
S. epidermidis   ------MAELPQSRINERNITSEMRESFLDYAMSVIVSRALPDVRDGLKPVHRRILYGLN  54
P. aeruginosa    ------MGELA-KEILPVNIEDELKQSYLDYAMSVIVGRALPDARDGLKPVHRRVLYAMS  53
E. coli          ------MSDLA-REITPVNIEEELKSSYLDYAMSVIVGRALPDVRDGLKPVHRRVLYAMN  53

M. tuberculosis  DSGFRPDPSHAKSARSVAETMGNYHPHGDASIYATLVRMAQPWSLRYPLVDGQGNFGSPG 120
S. aureus        EQGMTPDKSYKKSARIVGDVMGKYHPHGDSSIYEAMVRMAQDFSYRYPLVDGQGNFGSMD 120
S. epidermidis   EQGMTPDKPYKKSARIVGDVMGKYHPHGDSSIYEAMVRMAQDFSYRYPLVDGQGNFGSMD 114
P. aeruginosa    ELGNDWNKPYKKSARVVGDVIGKYHPHGDIAVYDTIVRMAQPFSLRYMLVDGQGNFGSVD 113
E. coli          VLGNDWNKAYKKSARVVGDVIGKYHPHGDLAVYDTIVRMAQPFSLRYMLVDGQGNFGSID 113

M. tuberculosis  NDPPAAMRYTEARLTPLAMEMLREIDEETVDFIPNYDGRVQEPTVLPSRFPNLLANGSGG 180
S. aureus        GDGAAAMRYTEARMTKITLELLRDINKDTIDFIDNYDGNEREPSVLPARFPNLLANGASG 180
S. epidermidis   GDGAAAMRYTEARMTKITLELLRDINKDTIDFIDNYDGNEREPSVLPARFPNLLVNGAAG 174
P. aeruginosa    GDNAAAMRYTEVRMAKLAHELLADLEKETVDWVPNYDGTEQIPAVMPTKIPNLLVNGSSG 173
E. coli          GDSAAAMRYTEIRLAKIAHELMADLEKETVDFVDNYDGTEKIPDVMPTKIPNLLVNGSSG 173

M. tuberculosis  IAVGMATNIPPHNLRELADAVFWALENHDADEEETLAAVMGRVKGPDFPTAGLIVGSQGT 240
S. aureus        IAVGMATNIPPHNLTELINGVLSLSKNPD----ISIAELMEDIEGPDFPTAGLILGKSGI 236
S. epidermidis   IAVGMATNIPPHNLTEVIDGVLSLSKNPD----ITINELMEDIQGPDFPTAGLVLGKSGI 230
P. aeruginosa    IAVGMATNIPPHNLGEVIDGCLALMDNPD----LTVDELMQYIPGPDFPTAGIINGRAGI 229
E. coli          IAVGMATNIPPHNLTEVINGCLAYIDDED----ISIEGLMEHIPGPDFPTAAIINGRRGI 229

M. tuberculosis  ADAYKTGRGSIRMRGVVEVE--EDSRGRTSLVITELPYQVNHDNFITSIAEQVRDGKLAG 298
S. aureus        RRAYETGRGSIQMRSRAVIE--ERGGGRQRIVVTEIPFQVNKARMIEKIAELVRDKKIDG 294
S. epidermidis   RRAYETGRGSIQMRSRAEIE--ERGGGRQRIVVTEIPFQVNKARMIEKIAELVRDKKIDG 288
P. aeruginosa    IEAYRTGRGRIYIRARAVVEEMEKGGGREQIIITELPYQLNKARLIEKIAELVKEKKIEG 289
E. coli          EEAYRTGRGKVYIRARAEVEV-DAKTGRETIIVHEIPYQVNKARLIEKIAELVKEKRVEG 288

M. tuberculosis  ISNIEDQSSDRVGLRIVIEIKRDAVAKVVINNLYKHTQLQTSFGANMLAIVDGVPRTLRL 358
S. aureus        ITDLRDETSLRTGVRVVIDVRKDANASVILNNLYKQTPLQTSFGVNMIALVNGRPKLINL 354
S. epidermidis   ITDLRDETSLRTGVRVVIDVRKDANASVILNNLYKQTPLQTSFGMNMIALVNGRPKLINL 348
P. aeruginosa    ISELRDES-DKDGMRVVIELRRGEVGEVVLNNLYAQTQLQSVFGINVVALVDGQPRTLNL 348
E. coli          ISALRDES-DKDGMRIVIEVKRDAVGEVVLNNLYSQTQLQVSFGINMVALHHGQPKIMNL 347

M. tuberculosis  DQLIRYYVDHQLDVIVRRTTYRLRKANERAHILRGLVKALDALDEVIALIRASETVDIAR 418
S. aureus        KEALVHYLEHQKTVVRRRTQYNLRKAKDRAHILEGLRIALDHIDEIISTIRESDTDKVAM 414
S. epidermidis   KEALIHYLEHQKTVVRRRTEYNLKKARDRAHILEGLRIALDHIDEIITTIRESDTDKIAM 408
P. aeruginosa    KDMLEVFVRHRREVVTRRTVYELRKARERGHILEGQAVALSNIDPVIELIKSSPTPAEAK 408
E. coli          KDIIAAFVRHRREVVTRRTIFELRKARDRAHILEALAVALANIDPIIELIRHAPTPAEAK 407
```

Figure S2. Alignment of beta-lactamase enzyme sequences from selected bacteria in the study. The gray regions correspond to non-similar and non-identical amino acids. The red regions correspond only to identical amino acids. The yellow regions are similar amino acids.

```
S. aureus        ---------------------------------MAKLLIMSVVSFCFIFLLLVF------  21
```

```
S. epidermidis ---------------------------------MAKLLIMSVVSFCFIFLLLVF------ 21
P. aeruginosa  -LVWARGFGYADRQH----RINASEHTAFHAGDLSKLLIASATLQLAERGQLSLDAPLQD 119
M. tuberculosis-TLYAKGFGVRDVGKGGGPDNKVDADTVFQLASVSKSVGATVVAHAVTDNVVTWDTPVVS 177
E. coli        RQRF--VYGVADVAS----QKANTLDTVYELGSMSKAFTGLVVQILIQEGRLRQGDDIIT 104

S. aureus      ------FRYI----------------------------LKRYFNYSLNYKVWYLTMLAGL 47
S. epidermidis ------FRYI----------------------------LKRYFNYSLNYKVWYLTVLAGL 47
P. aeruginosa  TLREFYVRSRFHADQSEADRAITFRRLLSHQSGLPGEHLPPLFGERPNSLGQLPAKVSGV 179
M. tuberculosisKLPWFALRDPYV------TGQVTIADLYSHRSGLPDHAGDLLEDLG--YDRRQVLQRLKY 229
E. coli        YLPEMRLNYQGK------PASLTVADFLYHTSGLPFSTLARLENPMPGSAVAQQLRNENL 158

S. aureus      IPFIPIKFSFIKFNNVNNQ-APTVE---SKSHDLNHNINTTKPIQEFTTDIHKFNWDSID 103
S. epidermidis IPFIPIKFSFIKFNNVNNQ-SPTVE---SRSHDLNHNINTTKPVQEFTTDIHKFNWDSID 103
P. aeruginosa  WLSNPPGTQVAHSNLGYELVGAAIERNTGKHFEQHMREHLLDPLQMTRSS---------- 229
M. tuberculosisLPLAPFRISYAYTNFGVTAAAEAVAAAAGQSWEDLSDEVLYRPLGMGSTS---------- 279
E. coli        LFAPGAKFSYASANYDVL--GAVIENVTGKTFTEVIAERLTQPLGMSATV---------- 206

S. aureus      NICTVIWIVLVIILSFKFLKALLYLKYLKKQSLYLNENEKNKIDTILFNHQYKKNIVIRK 163
S. epidermidis NICTVIWIVLVIILSFKFLKSLLYLKYLKRQSLYLNENEKNKIDTILFNHQYKKNIVIRK 163
P. aeruginosa  ---------------FAR-----------NAL-----------PQAQRAHGYSGG----- 247
M. tuberculosis---------------SRF-----------TDFLARPN---HAVNHVKVADRWEAR----- 305
E. coli        ---------------AVK-----------GDEIIVNK-----------ASGYKLG----- 224

S. aureus      AEAIQSPITFWYGKYIILIPSSYFKSVIDKRLKYI--ILHEYAHAKNRDTLHLIIFNIFS 221
S. epidermidis AETIQSPITFWYGKYIILIPSSYFKSVIDKRLKYI--ILHEYAHAKNRDTLHLIIFNIFS 221
P. aeruginosa  ---G-R------PGSASDLPVNDLWSSPVDLSRFVRMLFANGRHKERQ------------ 285
M. tuberculosis---YQR-------DPDAQSPAGGVSSSLNDMTHWLAMVLADGVYNGRR------------ 343
E. coli        ---FGKPVLFHAPLARNHVPAAYIHSTLPDMEIWIDAWLHRKALPATL------------ 269

S. aureus      IIMSYNPLVHIVKRKIIHDNEVEADRFVLNNINKNEFKTYAESIMDSVL------KTPFS 275
S. epidermidis IVMSYNPLIHIVKRKIIHDNEVEADRFVLNNINKNEFKTYAESIMDSVL------NIPFF 275
P. aeruginosa  -LLRKHSVEEMFRQQNAG-NAL---DFD-------CQVGLAWF-LSPCGSAPLEGGIRHY 332
M. tuberculosis-ITSPEALLPVYTPQVISRHPVSPRARA-------SFYGYGFN-VGVTSSGR----TEYS 390
E. coli        ----REAMSNSWRG--NSDVPLAADN-R-------ILYASGWF-IDQN---Q----GPYI 307
```

Figure S3. Alignment of PBP enzyme sequences from selected bacteria in the study. The gray regions correspond to non-similar and non-identical amino acids. The red regions correspond only to identical amino acids. The yellow regions are similar amino acids.

```
M. tuberculosis MVTKTT----------------LASATSGLLLLAVVAMSGCTPRPQGPGPAAEKFFAALA 44
S. aureus       ML--KRLKEKSNDEIVQNTINKRINFIFGVIVFIFAVLVLRLG--------------YLQ 44
S. epidermidis  ML--KRLKEKTNDEKMRNTMNKRINFIFGFIVFIFAIVVLRLG--------------YLQ 44
P. aeruginosa   ---------MKLNYFQGALYPWRFCVIVGLLLAMVGAIVWRIV--------------DLH 37
E. coli         MKAAAKTQKPKRQEEHANFISWRFALLCGCILLALAFLLGRVA--------------WLQ 46

M. tuberculosis IGDTASAAQLSDNPNEAREALNAAWAGLQAAHLDAQVLSAKYAEDTGTVAYRFSWHLPKD 104
S. aureus       IAQGSHYKQIIKNDENITV--------------NESVPRGRILDRNGK---VLVDNASKM 87
S. epidermidis  IAQGSHYKQLIKNDENITV--------------NESVPRGRILDRNGK---VLVDNASKM 87
P. aeruginosa   VIDHDFLKGQGDARSVRHI--------------AIPAHRGLITDRNGE---PLAVSTPVT 80
E. coli         VISPDMLVKEGDMRSLRVQ--------------QVSTSRGMITDRSGR---PLAVSVPVK 89

M. tuberculosis RIWTYDGQL-----------------KMA-------RDEGRWHVRWTTSGLHPKLGEHQT 140
S. aureus       AITYTRGRKTTQSEMLDTAEKLSKLIKMDTKKITERDKKDFWIQ------LHPKKAKAMM 141
S. epidermidis  SITYTRNRKTSQKEMLNTAKKLTDLIKMDTDKITERDKKDFWIQ------MYPSSAKKLM 141
P. aeruginosa   TLWAN------PKELM------------A--------AKERWPQ------LAAALGQ--- 105
E. coli         AIWAD------PKEVH------------DA---GGISVGDRWKA------LANALNI--- 119

M. tuberculosis ----FALR-----ADPPRRASVNEVGGTD--VLVP---GYLYHYSLDAGQAGRELFGTAH 186
S. aureus       TKEQAMLADGSIKQDQYDKQLLSKIGKSQLDELSSKDLQVLAIFRE--M----------- 188
S. epidermidis  RKEQLMLEDGSISQEQFDTQLRNKIGKKQLKQLTKKDLQVLAIYRE--M----------- 188
P. aeruginosa   ------------DTKLFA----DRIEQN-----AERE--FIYLVRG-------------- 128
E. coli         ------------PLDQLS----ARINAN-----PKGR--FIYLARQ-------------- 142

M. tuberculosis AVVGALHPFDDTLNDPQLLAEQASSSTQPLDLVTLHADDSNRVAAAIGQLPGVVITPQAE 246
S. aureus       --------NAGTVLDPQMIKNEDVSE-----------KEYAAVSQQLSKLPGVNTSMDWD 229
```

```
S. epidermidis   --------NAGSTLDPQTIKNEDVSE-----------KEYAAVSQQLSKLPGVNTTMDWD 229
P. aeruginosa    -------------LTPEQ-----------------------GEGVIALKVPGVYSIEEFR 152
E. coli          -------------VNPDM-----------------------ADYIKKLKLPGIHLREESR 166

M. tuberculosis  L-LPTD----------------------KHFAP--------AVLNDVKKAVVDELDGKAG 275
S. aureus        RKYPYGDTLRGIFGDVSTPAEGIPKELTEHYLSKGYSRNDRVGKSYLEYQYEDVLRGKKK 289
S. epidermidis   RKYPYGDTLRGIFGDVSTSTEGIPKELTEQYLSKGYSRNDRVGKSYLEYQYEDVLKGTKK 289
P. aeruginosa    RFYPAGEVVAHAVGF---------------------TDVDDRGREGIELAFDEWLAGVPG 191
E. coli          RYYPSGEVTAHLIGF---------------------TNVDSQGIEGVEKSFDKWLTGQPG 205

M. tuberculosis  WRVVSVNQNGVDVSVLHEV-APSPASSVSITLDRVVQNAA----QHAVNT---RG----G 323
S. aureus        EMKYTTDKSGKVTSSEVLN-PGARGQDLKLTIDIDLQKEVEALLDKQIKKLRSQGAKDMD 348
S. epidermidis   QMKYTTDKSGRVISSEVLN-PGSRGHDLQLTIDIDLQKKVESLLEKQISKLRSQGAKDMD 348
P. aeruginosa    KRQVLKDRRGRVIKDVQVTKNAKPGKTLALSIDLRLQYLAHRELRNALLE---NGA---K 245
E. coli          ERIVRKDRYGRVIEDISSTD-SQAAHNLALSIDERLQALVYRELNNAVAF---NKA---E 258
```

Figure S4. Alignment of CYP51 enzyme sequences from selected fungi in the study. The gray regions correspond to non-similar and non-identical amino acids. The red regions correspond only to identical amino acids. The yellow regions are similar amino acids. The black boxes represent the active site amino acids.

```
A. flavus        LVFHWIPFIGSTIHYGMDPYGFFFSCREKYGDIFTFILIGRPTTVYLGTQGNEFILNGKL 97
C. glabrata      LVFYWIPWVGSAIPYGTKPYEFFEDCQKKYGDIFSFMLIGRIMTVYLGPKGHEFIFNAKL 118
C. albicans      LVFYWIPWFGSAASYGQQPYEFFESCRQKYGDVFSFMLIGKIMTVYLGPKGHEFVFNAKL 109
C. tropicalis    MVFYWIPWFGSAASYGMQPYEFFEKCRLKYGDVFSFMLIGKVMTVYLGPKGHEFIYNAKL 109

A. flavus        KDVNAEEVYSPLTTPVFGSDVVYDCPNSKLIEQKKFIKFGLSQAALEAHVPLIEKEVEDY 157
C. glabrata      ADVSAEAAYSHLTTPVFGKGVIYDCPNHRLMEQKKFVKGALTKEAFVRYVPLIAEEIYKY 178
C. albicans      SDVSAEDAYKHLTTPVFGKGVIYDCPNSRLMEQKKFAKFALTTDSFKRYVPKIREEILNY 169
C. tropicalis    SDVSAEEAYTHLTTPVFGKGVIYDCPNSRLMEQKKFAKFALTTDSFKTYVPKIREEVLNY 169

A. flavus        LAMSPNFHG---TSGEVDIPAAMAEITIFTAGSALQGEEVRSKLTTEFAVLYHDLDKGFT 214
C. glabrata      FRNSKNFKINENNSGIVDVMVSQPEMTIFTASRSLLGKEMRDKLDTDFAYLYSDLDKGFT 238
C. albicans      FVTDESFKLKEKTHGVANVMKTQPEITIFTASRSLFGDEMRRIFDRSFAQLYSDLDKGFT 229
C. tropicalis    FVNDVSFKTKERDHGVASVMKTQPEITIFTASRCLFGDEMRKSFDRSFAQLYADLDKGFT 229

A. flavus        PINFMLPWAPLPHNKKRDAAHARMRSIYIDIINKRRNAGDNVPEKLDMIGNLMQCTYKNG 274
C. glabrata      PINFVFPNLPLEHYRKRDHAQQAISGTYMSLIKERREKNDIQ-NRDLIDELMKNSTYKDG 297
C. albicans      PINFVFPNLPLPHYWRRDAAQKKISATYMKEIKSRRDRGDIDPNRDLIDSLLIHSTYKDG 289
C. tropicalis    PINFVFPNLPLPHYWRRDAAQRKISAHYMKEIKRRRESGDIDPKRDLIDSLLVNSTYKDG 289

A. flavus        QPLPDKEIAHIMITLLMAGQHSSSSISSWIMLRLASQPAVVEELYQEQLANLERTGPNGS 334
C. glabrata      TKMTDQEIANLLIGVLMGGQHTSAATSAWCLLHLAERPDVQEELYQEQMRVLN-----ND 352
C. albicans      VKMTDQEIANLLIGILMGGQHTSASTSAWFLLHLGEKPHLQDVIYQEVVELLKEKG--GD 347
C. tropicalis    VKMTDQEIANLLIGVLMGGQHTSASTSAWFLLHLAEQPQLQDDLYEELTNLLKEKG--GD 347

A. flavus        LAPLQYKDFDNLPLHQNVIRETLRLHSSIHSLLRKVKNPLPVPGTPYVIPTSHVLLAAPG 394
C. glabrata      TKELTYDDLQNMPLLNQMIKETLRLHHPLHSLFRKVMRDVAIPNTSYVVPRDYHVLVSPG 412
C. albicans      LNDLTYEDLQKLPSVNNTIKETLRMHMPLHSIFRKVTNPLRIPETNYIVPKGHYVLVSPG 407
C. tropicalis    LNDLTYEDLQKLPLVNNTIKETLRMHMPLHSIFRKVMNPLRVPNTKYVIPKGHYVLVSAG 407
```

Figure S5. Alignment of SAP-2 enzyme sequences from selected fungi in the study. The gray regions correspond to non-similar and non-identical amino acids. The red regions correspond only to identical amino acids. The yellow regions are similar amino acids.

```
C. albicans      -----MFLKNIFIALAIALLVDATPTTT-----------------KRSAGFVALDFSVVK 38
C. tropicalis    MVWVIGLMKQVFISIVFGLTVEGLAISKLNDRGAAPGLMGNFFDKRSTPGVLSLELYVNR 60

C. albicans      TPKAFPVTNG----QEGKTSKRQAVPVTLHNEQVTYAADITVGSNNQKLNVIVDTGSSDL 94
C. tropicalis    NHDDSNFTIGPHFVVNEYSKRDDYISVELYNEQVTYSANFTVGSNSQKQNVIVDTGSSDL 120

C. albicans      WVPDVNVDCQVTYSDQTADFCKQKGTYDPSGSSASQDLNTPFKIGYGDGSSSQGTLYKDT 154
```

```
C. tropicalis    WVVDSSANCQEK-SGYSSDYCFSGGTYDPSSSSTIQELGKSFNIRYGDGSSSSGTWVKDT 179

C. albicans      VGFGGVSIKNQVLADVDSTSIDQGILGVGYKTNEAG-GSYDNVPVTLKKQGVIAKNAYSL 213
C. tropicalis    VGINGAIILNQQFGDVNSTSVSQGILGIGLDTNESTDTIYENFPINLKEQGFINTNAYSL 239

C. albicans      YLNSPDAATGQIIFGGVDNAKYSGSLIALPVTSDRELRISLGSVEVSGKTINTDNVDVLV 273
C. tropicalis    YLNAPSATSGTIIFGGIDHAKYTGSLTTLPLTSNREFTIQTNSATVGTSTID-INTGLLL 298

C. albicans      DSGTTITYLQQDLADQIIKAFNGKLTQDSNGNSFYEVDCNLSGDVVFNFSKNAKISVPAS 333
C. tropicalis    DSGTTLTYLPQSVVDSIANAIGGDITYNRP-IGAYIWSCNRNGKVTYNFPQGLNIDIPYS 357

C. albicans      EFAASLQGDDGQPYDKCQLLF-DVNDANILGDNFLRSAYIVYDLDDNEISLAQVKYTSAS 392
C. tropicalis    DLAVPLYYSNGAVAGFCALGILYGENFNILGDNFLRHAYVVYNLDALTISLAPVVYTSDS 417

C. albicans      SISALT    398
C. tropicalis    NVTIV-    422
```

Figure S6. Alignment of DHFR enzyme sequences from selected fungi in the study. The gray regions correspond to non-similar and non-identical amino acids. The red regions correspond only to identical amino acids. The yellow regions are similar amino acids.

```
C. glabrata      -MSK-VPVVGI---------VAALLPEMGIGFQGNLPW-RLAKEMKYFREVTTLTNDNSK  48
C. albicans      -MLK-PNVAII---------VAALKPALGIGYKGKMPW-RLRKEIRYFKDVTTRTTKPNT  48
C. tropicalis    MQSK-PVISII---------VAALKPSLGIGNNGKMPW-RLRKEIRYFKDVTSKTTVENS  49
A. flavus        MP-PTNPLTLIVATTPIPTRE---KTLLGIGLNGTLPWPRIKADMSFFARVTTRPPRPGT  56
T. rubrum        MPAKLPPLTLVVATTPITTPTNPGILKLGIGKEGTLPWPRIKKDMSFFARVTTRPPATAT  60

C. glabrata      --------QNVVIMGRKTWESIPQKFRPLPKRINVVVSRSFDGELRKVEDG---------  91
C. albicans      --------RNAVIMGRKTWESIPQKFRPLPDRLNIILSRSYENEII--DDN---------  89
C. tropicalis    --------RNAVIMGRKTWESIPVKFRPLPDRLNIILSRSYENEII--DEN---------  90
A. flavus        --------TNAMIMGRKTYDSVPKSLRPLGKRINVIVTRDVEGVSKRVAEE-LKEKR--- 104
T. rubrum        ASGSASPAINAVIMGRKTYDSIPAKFRPLSKRLNVIITRDESGSVKERAIADWNASRKRE 120

C. glabrata      ----------------------------IYHSNSLRNCLTALQSSLA---------NENK 114
C. albicans      ----------------------------IIHASSIE-------SSLN---------LVSD 105
C. tropicalis    ----------------------------IIHANSVE-------SSLS---------FMSN 106
A. flavus        ----AKMAAAAAAATSAGENKEEGPITDAIVSSGLEAALEDVEEKF-----------KGG 149
T. rubrum        LEKQADQDTDKAAATST-----STEEPEVIVSSSLEDALSTLQRNFVISSSSDVQGGKKR 175

C. glabrata      IERIYIIGGGEIYRQSMD--L-------ADHWLITKIMPL----------PETTIPQMDT 155
C. albicans      VERVFIIGGAEIYNELINNSL-------VSHLLITEIE-H----------PSPESIEMDT 147
C. tropicalis    IDRVFIIGGAEIYNDLINNPL-------VTQLLITEIE-H----------SNPESVEMDT 148
A. flavus        LGSVFVIGGAEIYATALG-----LGGDRPVRIVMTNVEKKGV-------DGEKAVFECDT 197
T. rubrum        LGNVYIMGGSEIYASSLRLTADALGENNPLRIVMTDIRRRADGNAQCDVEDLVDGFECDT 235

C. glabrata      FLQKQ----ELEQRFYDNSDKLVDFLPSSIQLEERLTSQEWNGELVKGIPVQEKGYQFYF 211
C. albicans      FLKFP-----LESWTKQPKSELQKFVGDTV-LEDD---------------IKEGDFTYNY 186
C. tropicalis    FLKFP-----LEKWTKQPKSELQKFIGDEITLEDN---------------INEGDFTYNY 188
A. flavus        FFPIDEELLMEKGWRKVSAEEVTEWVGEPVSGE-----WKDEGE-----------VRIQM 241
T. rubrum        CFPLDGKG-LKEGWNKVPSEKLAEWVGEAVSSD-----WTWEGD-----------IAMKI 278
```

Figure S7. Ramachandran plot of homology models generated for (A) *S. epidermidis* DNA gyrase, (B) *S. epidermidis* beta-lactamase, (C) *S. epidermidis* PBP, (D) *S. tropicalis* SAP-2, (E) DHFR from *S. tropicalis*, (F) CYP51 from *A. flavus* and (G) DHFR from *T. rubrum*. The colored regions represent the allowed and favored regions of secondary structures and the white regions represent the forbidden regions.

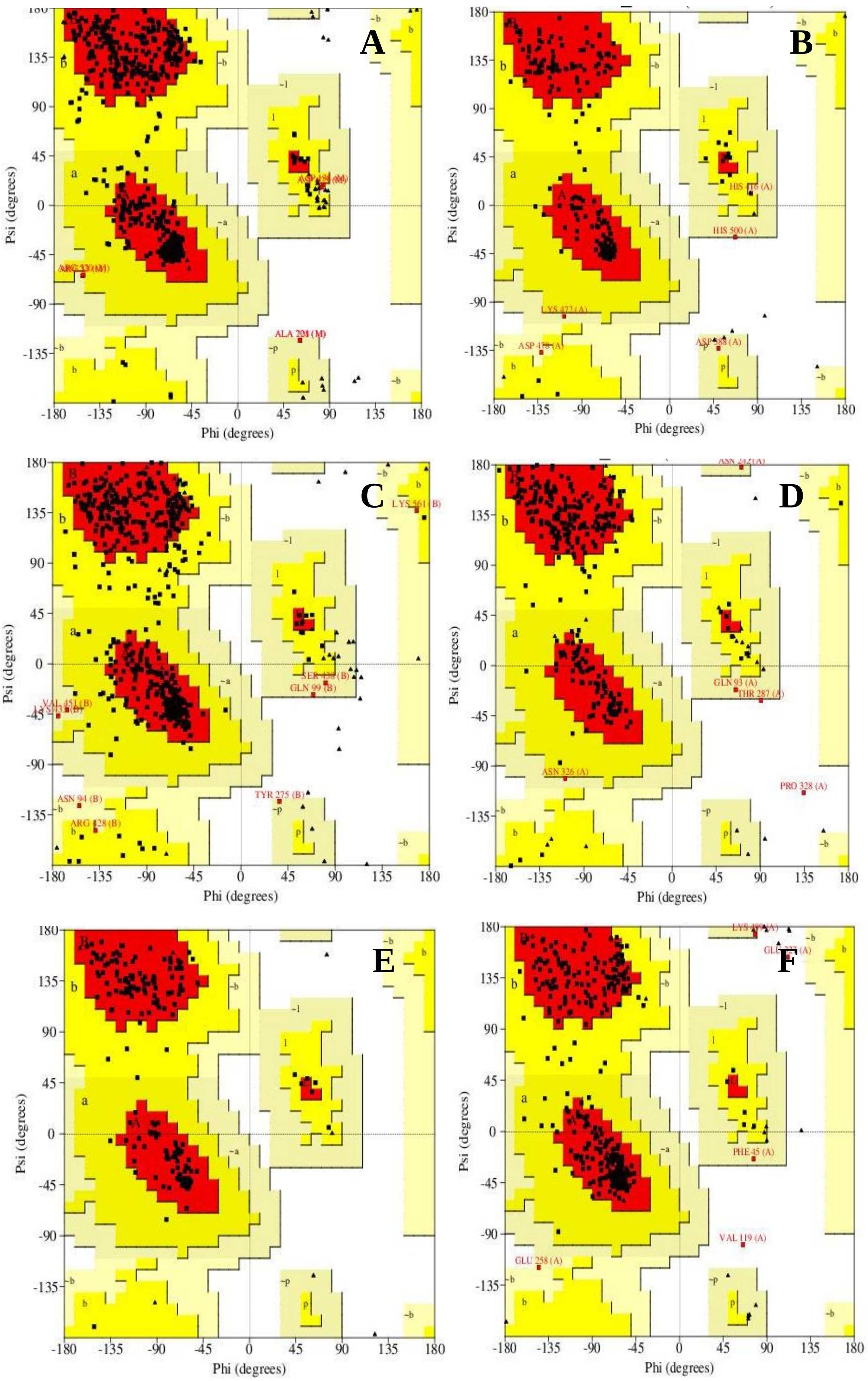

A
B
C
D
E
F
Psi (degrees)
Phi (degrees)
HIS 500 (A)
LYS 472 (A)
ASP 478 (A)
LYS 561 (B)
GLN 99 (B)
ASN 94 (B)
ARG 128 (B)
TYR 275 (B)
GLN 93 (A)
THR 287 (A)
ASN 326 (A)
PRO 328 (A)
PHE 45 (A)
VAL 119 (A)
GLU 258 (A)

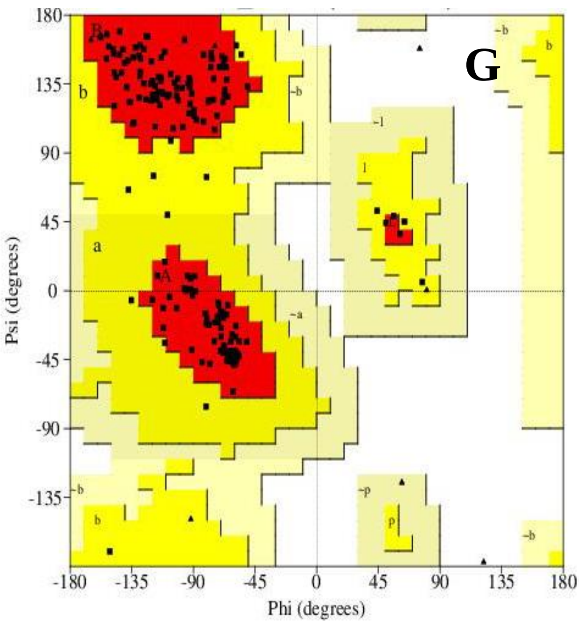
G
180
135
90
45
0
-45
-90
-135
Psi (degrees)
-180
-135
-90
-45
0
45
90
135
180
Phi (degrees)
B
b
a
A
~b
~a
l
~l
~p
p
